# Spiritual care competency and its relationship with clinical self-efficacy in nursing students

**DOI:** 10.1186/s12909-023-04937-3

**Published:** 2023-12-08

**Authors:** Zahra Sahebi, Maasoumeh Barkhordari-Sharifabad

**Affiliations:** Department of Nursing, School of Medical Sciences, Yazd Branch, Islamic Azad University, Yazd, Iran

**Keywords:** Clinical self-efficacy, Spiritual care competency, Spirituality, Nursing students

## Abstract

**Background:**

Spiritual care occupies a special place in holistic care and improving the quality of services provided to patients. The component of clinical self-efficacy is recognized as a prerequisite for clinical capacity and competency. The current study determined the level of spiritual care competency and its relationship with clinical self-efficacy in nursing students.

**Methods:**

This cross-sectional study was conducted on 194 nursing students studying in medical sciences universities in Yazd, Iran, who were selected by convenient sampling. Data collection tools were a spiritual care competency scale and nursing students’ clinical self-efficacy questionnaire. The gleaned data were analyzed by SPSS20 using descriptive and inferential statistics.

**Results:**

The mean scores of spiritual care competency and clinical self-efficacy of students were 70.29 ± 9.58 and 102.40 ± 21.57, respectively. The results of Pearson’s correlation coefficient test showed that clinical self-efficacy had a positive significant correlation with spiritual care competency (*p* = 0.04, r = 0.14). The mean score of spiritual care competency was higher in female students (71.10 ± 9.81) than male students (67.50 ± 8.23) with a statistically significant difference (*p* < 0.05). The regression test results suggested that clinical self-efficacy can be considered as a predictor of spiritual care competency.

**Conclusion:**

The results revealed that spiritual care competency in nursing students was at an average level, and the higher the level of clinical self-efficacy is, the more developed the spiritual care competency will be. Thus, nursing educators and health system managers should pay special attention to improving the clinical self-efficacy of nursing students to develop their spiritual care competency.

**Supplementary Information:**

The online version contains supplementary material available at 10.1186/s12909-023-04937-3.

## Background

The role of spirituality in promoting patient health has recently attracted much attention [1]. Spirituality has several definitions, most of which deal with the ultimate goal in life, the experience of a transcendent dimension that gives meaning to existence and the capacity to experience the sacred [2]. Spirituality is an inner resource that directly contributes to a sense of purpose in life, resilience, adaptive coping, and well-being [3]. Altruism, interconnectedness, love, contemplative practice, and religious and spiritual reflection constitute the dimensions of spirituality [4].

In the Iranian context, religious values, beliefs and practices have given new meaning to the professional life of nurses, and the spiritual foundation and religious beliefs of nurses help them give meaning to care in a positive way [5]. Islam is the dominant religion in Iran [6], and spirituality in Islam means having human values such as faith in God, respect, acceptance of others, piety, serving the people, optimism, and honesty [7]. Helping fulfill the spiritual needs of patients and their families is considered an essential element of clinical care [8], and providing spiritual care is an important part of nurses’ performance [9].

Competency in spiritual care refers to the ability of nurses to assess and provide measures to meet the spiritual needs of patients in cooperation with the multidisciplinary healthcare team [10]. Research findings have indicated that the concept of spirituality and spiritual care in the health system still remains a subjective, ambiguous, and complex idea [11]. They also show that many nurses have not received training in spirituality and spiritual care and consider their ability to provide spiritual care to be weak [12]. One study found that nurses do not have proper professional qualifications in providing spiritual care services [13], and role models do not play a sufficient role at the bedside to provide spiritual care [14].

Self-efficacy is necessary for providing comprehensive and holistically valuable care [11]. Clinical self-efficacy is nurses’ judgments about the ability to organize and administer nursing care independently based on the nursing process at the bedside [15]. The results of studies indicate that self-efficacy has a positive relationship with clinical skill competency [16], self-esteem and sense of belonging in the clinical setting [17], creativity [18], clinical performance [19], work commitment [20], and self-regulated learning [21].

Nursing students are the would-be nurses of the future who will have to practice spiritual care in clinical practice. Hence, their competencies in spiritual care greatly affect the quality of their patients’ spiritual care. The clinical education of nursing students predisposes them to applying nursing science at the bedside [22], and improving clinical self-efficacy leads to increasing the quality of performance of the workforce trained for nursing [23]. Based on the information stated above, the current study aimed to determine the level of spiritual care competency and its relationship with clinical self-efficacy in nursing students.

## Methods

The participants in this cross-sectional study were selected from nursing schools in Yazd province, Iran, from April 2022 to June 2022. The research sample was considered to be 176 nursing students, with a confidence interval of 95%, test power of 80%, and correlation coefficient of 0.21, based on the pilot study. With a subject attrition rate of 10%, the sample volume was calculated as 194 participants. Sampling was done using the convenient sampling method. Participants were selected based on the following criteria: (a) being a fourth-year nursing student, (b) engaged in undergraduate studies, (c) studying in the second semester of the 2021/2022, academic year, (d) no history of physical or mental problems, and (e) willing to participate in the study. This study used three questionnaires to collect data.

### Demographic characteristics questionnaire

This collected information on the age, gender, marital status, and grade point average of the participants.

### Spiritual care competency scale

Developed by Van Leeuwen et al. (2009), [24], this scale contains 27 items covering 6 dimensions: “evaluation and implementation of spiritual care” (items 1–6), “specialization and improvement of the quality of spiritual care” (items 7–12), “individual support and consultation with the patient” (items 13–18), “referral to specialists” (items 19–21), “attitude towards the patient’s spirituality” (items 22–25), and “communications” (items 26–27). It is scored on a 5-point Likert scale (completely agree = 5 to completely disagree = 1). The total score of this scale varies between 27 and 135; scores of 27 to 62, 63 to 98, and above 98 indicate low, medium, and high competency, respectively. Construct validity was examined through exploratory factor analysis by Van Leeuwen et al., and the results showed that these six dimensions accounted for 53% of the total variance. The reliability of the scale was calculated using Cronbach’s α coefficient, which was equal to 0.82, indicating appropriate reliability [24]. After translating the scale, Khalaj et al. investigated its validity and reliability on nursing students. Their results indicated that the psychometric quality of the Persian version of the scale was satisfactory. They reported a Cronbach’s α coefficient of 0.77 for the whole instrument and between 0.65 and 0.85 for the subscales [25]. In the present study, Cronbach’s α coefficient was 0.83.

### Nursing students’ clinical self-efficacy questionnaire

This questionnaire was created by Cheraghi et al. (2009) [15]. This tool has 37 items and is graded on a 4-point Likert scale (from not sure at all = 1 to completely sure = 4). The minimum and maximum points for participants are 37 and 148. Higher scores indicate higher levels of clinical self-efficacy. The total score of the instrument is classified into three categories: low (37–74), medium (74.1–111), and high (111.1–148). The content validity of the tool was reviewed and confirmed by twenty faculty members of the school of nursing. A Cronbach’s α coefficient of 0.96 and test-retest correlation coefficient of 0.73 have been reported [15]. In the present study, Cronbach’s α was 0.94.

Data were analyzed with SPSS20 using descriptive statistics (absolute and relative frequency, mean and standard deviation) and inferential statistics (parametric t-test, analysis of variance, (ANOVA), Pearson correlation coefficient, and regression). The Kolmogorov-Smirnov (KS) test was run to confirm the assumption of normality of data distribution (P > 0.05).

## Findings

All of the 194 distributed questionnaires were completed and analyzed. Table 1 presents the demographic characteristics of the participants.

**Table 1:** Demographic Characteristics of Participants

Variable		F (%)
Gender	Male	44 (22.7)
	Female	150 (77.3)
Marital status	Single	173 (89.2)
	Married	21 (10.8)
**Variable**		**M ± SD**
Age		21.68 ± 2.30
Grade point average		17.02 ± 0.95

The mean scores of spiritual care competency and clinical self-efficacy were 70.29 ± 9.58 and 102.40 ± 21.57, respectively. Based on the standardized scores, the highest mean of spiritual care competency was related to the area of communication (58.16 ± 25.18) and the lowest mean was related to the area of specialization and improving the quality of spiritual care (44.72 ± 18.44) (Table 2). The majority of participants had an average level of spiritual care competency (81.5%) and clinical self-efficacy (56.7%).

**Table 2:** Mean and standard deviation of spiritual care competency and clinical self-efficacy in participants

Variables		M ± SD	Standardized scores based on percentage
**Spiritual care competency**	Evaluation and implementation of spiritual care	15.74 ± 2.91	54.15 ± 16.15
	Specialization and improving the quality of spiritual care	14.60 ± 3.13	44.72 ± 18.44
	Individual support and counseling of patients	15.62 ± 2.99	53.46 ± 16.95
	Referral to specialists	7.07 ± 1.81	45.24 ± 20.12
	Attitude about patients’ spirituality	11.76 ± 2.32	52.90 ± 25.78
	Communications	5.48 ± 1.51	58.16 ± 25.18
	Total	70.29 ± 9.58	39.19 ± 15.45
**Clinical self-efficacy**		102.40 ± 21.57	-

The results of Pearson’s correlation coefficient test demonstrated that clinical self-efficacy had a positive significant relationship with spiritual care competency (*p* = 0.041, r = 0.147). The relationships between clinical self-efficacy with the dimension of “evaluation and implementation of spiritual care” (*p* = 0.013, r = 0.178) and the dimension of “specialization and improving the quality of spiritual care” (*p* = 0.048, r = 0.142) were also positive and significant (Table 3).

**Table 3:** Correlation between ‘spiritual care competency and its dimensions’ and ‘clinical self-efficacy’

Variables	Clinical self-efficacy	
	Correlation coefficient	*p*-value
Evaluation and implementation of spiritual care	0.178	0.013
Specialization and improving the quality of spiritual care	0.142	0.048
Individual support and counseling of patients	0.016	0.828
Referral to specialists	0.061	0.396
Attitude about patients’ spirituality	0.133	0.064
Communications	-0.017	0.813
Spiritual care competency (total)	0.147	0.041

No statistically significant relationship was observed between “spiritual care competency” and “age, grade point average, or marital status” (*p* > 0.05). The mean score of spiritual care competency was higher in female students (9.817 ± 71.1) than male students (8.233 ± 67.5), a significant difference according to the independent t-test (*p* < 0.05).

Simultaneous linear regression analysis was used to investigate the role of clinical self-efficacy and demographic variables (as a predictor variable) in spiritual care competency (as a dependent variable). Among the demographic variables, only gender, which had a significant relationship with spiritual care competency, was included in the regression analysis. The results indicated that the multiple correlation coefficient (R) was 0.212 and the squared coefficient (R^2^) was 0.045. The results of ANOVA [*p* = 0.012, F = 4.50 (191, 2)] showed that this value of R^2^ was significant (Table 4).

**Table 4:** Summary of regression model results

Predictor Variable	Unstandardized coefficient		Standardized coefficient	t-observed	*p* -value
	B	SE	β		
Fixed	61.062	3.493		17.483	< 0.001
Clinical self-efficacy	0.064	0.031	0.144	2.031	0.044
Gender (reference: male)					
Female	3.495	1.614	0.153	2.166	0.032
Adjusted R^2^ = 0.035	R^2^ = 0.045			R = 0.212	

Dependent variable: Spiritual care competency

## Discussion

The goal of the present study was to determine the level of spiritual care competency and its relationship with clinical self-efficacy in nursing students.

The present study found the level of spiritual care competency to be at an average level, which was consistent with the results of other researchers [26, 27], although it contrasted with the results of Jafari and Fallahi-Khoshknab [28] and Nasehi et al. [29]. Kalkim et al. reported that the level of spiritual care competency of nursing students in Turkey was not optimal [30]. Adib-HajBagheri and Zehtabchi found that nurses did not have appropriate professional competency in providing spiritual care and had not yet received training related to this concept [12]. Study results also indicate the need to pay more attention to spiritual care competency in students and to discover many strategies to improve the spiritual care competency of nursing students. Iran’s nursing curriculum neglects spiritual care, and the educational syllabi need to be revised in order to improve nursing students’ understanding of spiritual care and how to use it in clinical practice.

The results further revealed an average level of spiritual care competency in almost all areas. The highest and lowest means pertained to the areas of “communications” and “specialization and improving the quality of spiritual care”, respectively. These results are inconsistent with the findings of other studies [12, 31]. In the study by Yazdanparast et al., nursing students scored high in most areas of spiritual care [32]. The area of “specialization and improving the quality of spiritual care” includes those activities of nurses that aim to ensure quality and develop policy in the field of spiritual care. In fact, this area refers to participation at the institutional level that goes beyond the primary care process and through which nurses contribute to the promotion of professional performance [24]. This area had the lowest mean score in the present study. Recognizing and using specialization strategies such as teamwork, dynamic learning, and improving students’ self-concept can be helpful in improving this area [33], which, given its low level in the present study, should be taken into consideration by nursing educators.

The results also indicated that clinical self-efficacy was at an average level in the majority of research units, which is consistent with the findings of Salimi et al. [34]. Inconsistent with these results, the findings of some studies have indicated that students had a high level of self-efficacy [17, 35, 36], although Zhang et al. reported the level of self-efficacy of Chinese students as low [37]. Considering the many factors affecting the self-efficacy of nursing students such as academic semester, type of education and clinical training, achieving different results of self-efficacy levels in different studies is predictable and inevitable [17, 38, 39].

The findings of the current research showed that the higher the clinical self-efficacy is, the more developed the spiritual care competency in students will be, and that spiritual care competency can be predicted by clinical self-efficacy. Haghani et al. also concluded that the higher the clinical self-efficacy is, the better the performance of nurses in different dimensions of care will be [40]. Chang et al. also found that self-efficacy had a significant positive relationship with the spiritual care competency of nursing students in China [41]. The findings of Cheraghi et al. also suggested that there was a positive significant correlation between “clinical self-efficacy” and “clinical nursing performance” [19]. In Opacic’s study, it was also found that the clinical self-efficacy of final year medical students had a significant positive relationship with their performance at the patient’s bedside, and students with high self-efficacy were more inclined to improve the quality of their clinical performance [42]. Moreover, Goldenberg et al. reported a significant positive relationship between self-efficacy and the nursing process, including patient examination, planning, implementation, and program evaluation [43].

It can be said that students who have higher clinical self-efficacy have a better understanding of the concept of “holistic care” and attend to the spiritual dimension of the patient in addition to other dimensions [44]. Put another way, students with higher self-efficacy attempt to provide related spiritual care and develop their spiritual care competency in addition to improving their professional knowledge and skills.

Furthermore, the results indicated that with an increase in clinical self-efficacy, the mean score of the areas of “evaluation and implementation of spiritual care” and “specialization and improving the quality of spiritual care” also increased, but this relationship was not significant for other dimensions. The results of Chang et al.‘s study showed that self-efficacy is related to all of the dimensions of spiritual care competency of nursing students in China [41]. Of course, in Chang et al.‘s study, general self-efficacy was evaluated, while the present study evaluated clinical self-efficacy, which includes the items of patient examination, nursing diagnosis, and planning, implementation, and evaluation of the care plan. Students with higher clinical self-efficacy are better able to determine patients’ spiritual needs and/or problems, plan spiritual care, and contribute to the promotion of professional performance by participating in quality assurance activities and policy development in the field of spiritual care.

## Limitations of the study

One of the limitations of the present study was its cross-sectional nature, which makes it difficult to make a causal inference. Moreover, the questionnaires were of the self-report type, which creates the possibility of social desirability bias. To resolve this limitation, the authors tried to thoroughly explain the study objectives to the students and to assure participants that the information they gave would remain confidential and anonymous and the reports would be presented in general.

### Recommendations for future research

For future research, it is suggested that studies investigate the development and implementation of focused interventions aimed at improving the clinical self-efficacy of nursing students and its effect on spiritual care competence. These interventions might encompass different educational methods such as problem-based learning, mentorship programs, etc. [45]. Since spirituality is a subjective and culture-oriented term [46], it is recommended that multicenter studies with larger samples be conducted in educational settings with different cultures and educational contexts. Engaging in longitudinal investigations that track nursing students over the course of their education can provide valuable insights into the progression of their spiritual care competency and clinical self-efficacy. A comparative study between the nursing categories could help provide evidence of similarities or differences in the mentioned variables. It is also suggested that the relationship between spiritual care competency and other variables such as awareness of bio-ethical principles, organizational commitment, and responsibility should be investigated.

## Conclusion

The results of the current study showed that spiritual care competency in nursing students was at an average level, and with the improvement of clinical self-efficacy, spiritual care competency is also developed. Thus, nursing educators and health system managers should pay special attention to methods that can improve the clinical self-efficacy of nursing students to improve their perceived competency in spiritual care. Theoretical training and clinical practice in caring for patients with refractory diseases and implementing innovative and active educational strategies are potential methods for developing clinical self-efficacy and ability in spiritual care.

### Electronic supplementary material

Below is the link to the electronic supplementary material.

Supplementary Material 1

Supplementary Material 2

## Data Availability

The datasets generated and analyzed during the current study are not publicly available due to an agreement with the participants on the confidentiality of the data but are available from the corresponding author on reasonable request.

## Abbreviations

SD: Standard Deviation
KS: Kolmogorov-Smirnov
ANOVA: Analysis of variance
