# Supplementary material for: Spiritual care competency and its relationship with clinical self-efficacy in nursing students
Source: BMC Med Educ. 2023 Dec 8;23:937. doi: 10.1186/s12909-023-04937-3 (PMC10709853; doi:10.1186/s12909-023-04937-3)
Supplement: Supplementary file 1 — Supplementary Material 1 [file 12909_2023_4937_MOESM1_ESM.docx]

Dear student

Greetings and wishing you well

Respectfully, while thanking you for your consent to participate in this research, "Spiritual care competency and its relationship with clinical self-efficacy in nursing students", please carefully fill out the following questionnaires. It is reminded again that all information remains confidential and the data will be used to improve the clinical education of nursing students.

***Demographic Characteristics Questionnaire***:

Age: ........................ years

Gender: Male 🞏 Female 🞏

Marital status: Single 🞏 Married 🞏

Grade point average: …………………
